# Advances and Prospects of Wearable Ultrasound Devices

**DOI:** 10.3390/mi17040419

**Published:** 2026-03-30

**Authors:** Matthew Xinhu Ren, Tyler Ngo, Xunan Liu, Leopard Liu, Yushun Zeng, Qifa Zhou

**Affiliations:** 1 Alfred E. Mann Department of Biomedical Engineering, Viterbi School of Engineering, University of Southern California, Los Angeles, CA 90089, USA; 2 USC Roski Eye Institute, Keck School of Medicine, University of Southern California, Los Angeles, CA 90033, USA

## 1. Introduction

Conventional ultrasound imaging relies on sound waves transmitted into the body via ultrasound transducers. These waves propagate through tissues and reflect as echoes at interfaces between materials of different acoustic impedance. The pulse-echo technique processes these reflected signals in real time to construct two- or three-dimensional anatomical images based on time-of-flight and amplitude differences [1,2,3]. Additionally, the Doppler effect enables the measurement of blood flow velocity by detecting frequency shifts in echoes from moving scatterers, such as red blood cells [4,5,6]. These capabilities have established ultrasound as a cornerstone of non-invasive medical diagnostics, offering real-time visualization, no ionizing radiation, greater portability than modalities such as magnetic resonance imaging (MRI), and cost-effectiveness across a wide range of biomedical applications [7].

Despite these advantages, traditional ultrasound systems face significant limitations that hinder their integration into broader modern healthcare paradigms that emphasize continuous and personalized monitoring. Conventional probes are typically bulky and rigid, requiring handheld operation and connection to a large back-end system [7,8,9]. Image acquisition and interpretation remain highly operator-dependent, relying on skilled technicians to operate the probe correctly. This introduces variability, limits accessibility in resource-constrained or remote settings, and restricts examinations to short, episodic clinical sessions [10,11,12,13,14]. These constraints have driven the development of wearable ultrasound devices, offering continuous, operator-independent monitoring capabilities essential for real-time diagnostics [15,16]. By conforming to the skin surface and eliminating manual positioning, these devices enable longitudinal tracking of physiological parameters without compromising patient mobility or requiring specialized personnel, thereby supporting proactive interventions in disease progression and home-based care [17,18,19].

In recent years, wearable ultrasound has advanced rapidly, from early flexible transducers to breakthroughs in stretchable arrays, bioadhesive integration, flexible interconnects, and autonomous operation [20,21,22,23]. This review highlights these transformative developments in device architecture, fabrication techniques, imaging modalities, and biomedical applications while addressing current challenges and future directions for clinical translation and multimodal functionality.

## 2. Classification of Advanced Wearable Ultrasound

**Figure 1:**
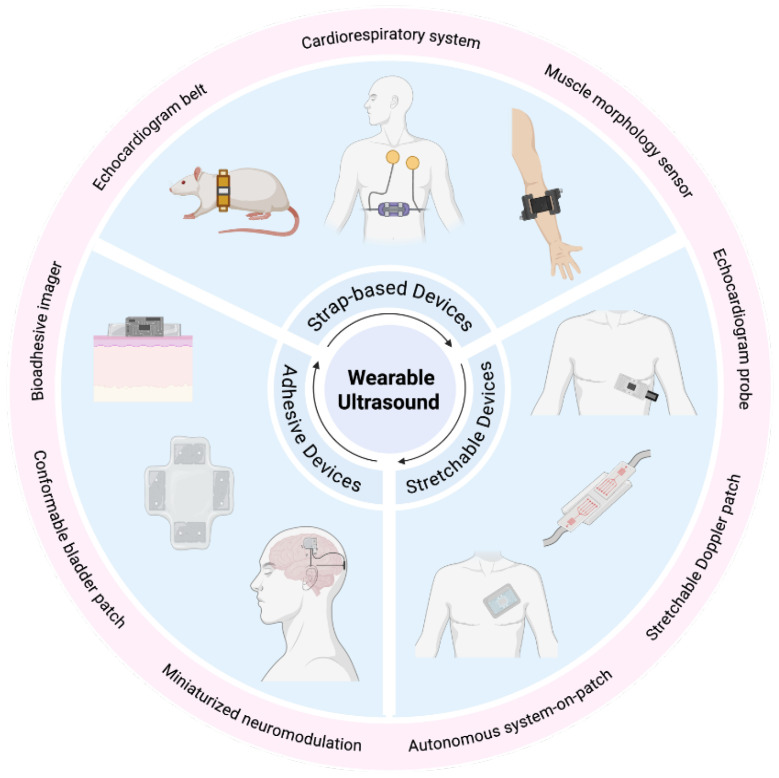
Representative recent wearable ultrasound devices, categorized by wearability and functionality for different target organs.

Wearable ultrasound devices can be classified into three primary types based on their mechanical properties, conformability, and wearable strategies: strap-based, adhesive, and stretchable. This classification reflects the evolution of conventional technology to fully soft, skin-conformable devices, each offering distinct trade-offs in imaging performance, comfort, and durability (Figure 1) [23,24].

### 2.1. Strap-Based Wearable Ultrasound

Strap-based devices typically incorporate miniaturized conventional transducers, made with either dense piezoelectric elements or capacitive micromachined ultrasound transducers (CMUTs), with substrates such as printed circuit boards [25,26]. Wearability is achieved through flexible straps that adhere to the body. These designs preserve high acoustic performance, including superior spatial resolution, broad bandwidth, and robust beamforming, due to optimized acoustic stacks and stable element positioning [26,27]. However, the rigidity of the dense transducer restricts adaptation to curved or dynamically deforming body surfaces, often requiring additional acoustic coupling gel and increasing susceptibility to motion artifacts [28,29].

Zeng et al. developed a wearable ultrasound array belt (WUAB) for real-time echocardiography in smaller mammal models [30]. The device features a rigid 20 MHz 1–3 composite lead zirconate titanate (PZT) array on a flexible printed circuit board (FPCB) belt strap tailored for smaller rodents. B-mode echocardiograms of the left ventricle are used to track ventricular dimensional changes, while extracted M-mode images provide detailed assessments of mechanical activity throughout the cardiac cycle [30]. Vostrikov et al. reported a wearable ultra-low-power ultrasound (WULPUS) system for simultaneous echocardiographic and respiratory monitoring [31]. Using a 2.25 MHz, 8-channel rigid transducer secured via chest straps in the parasternal window, the system generates M-mode representations from A-mode scans to extract respiration-induced scatterer motion. Despite these capabilities, limitations in wearability and mechanical robustness persist, as linear array-based probes constrain further device miniaturization compared with stretchable alternatives [32].

For muscular monitoring, Yang et al. proposed a wearable multichannel A-mode ultrasound system (WMAUS) with eight miniaturized rigid PZT transducers arranged around the forearm via a customized armband [33]. The rigid composite and optimized matching layer enable high excitation pulses and superior detection depth. Shorter pulses provide greater axial resolution, enabling efficient detection of muscle shape and deformation for applications in prosthesis control and clinical rehabilitation. However, the rigid transducers lack stability and conformability to the natural contours of the forearm, leading to potential device misplacement, signal degradation, and discomfort during prolonged use [33,34,35].

### 2.2. Adhesive Wearable Ultrasound

Adhesive devices integrate adhesive substrates, such as hydrogel or polydimethylsiloxane (PDMS), with rigid or flexible thin-film piezoelectric materials, thereby providing robust skin adhesion and moderate curvature conformity [36,37]. This combination improves skin contact, reduces gel requirements, and enhances patient comfort during extended wear compared with rigid systems. Although substrate compliance may introduce minor perturbations in acoustic efficiency, recent optimizations in acoustic matching and backing layers have substantially alleviated these effects [38].

Wang et al. presented a bioadhesive ultrasound device (BAUS) combining a high-density rigid piezoelectric array with a flexible bioadhesive hydrogel couplant [39]. The thin couplant is primarily composed of a polyacrylamide-chitosan interpenetrating polymer network encapsulated by an elastomer member and coated in a bioadhesive layer, offering strong adhesion and long-term stability. However, pitch-induced grating lobes and poor elevational resolution due to the lack of elevation focusing constrain the device’s imaging quality and depth [40,41]. Similarly, Zhang et al. designed a conformable ultrasound bladder patch (cUSB-Patch) for real-time bladder volume imaging [42]. It comprises five individual one-dimensional (1D) phased arrays with local rigidity embedded in flexible silicone adhesion rubber, enabling global malleability, consistent skin contact, and significant mechanical deformation. This design configuration delivers a wider multi-axial field of view (FOV) for full organ imaging without manual rotation.

For long-term neuromodulation applications, Tang et al. introduced a miniaturized bioadhesive-coupled ultrasound transducer (MiniUlTra) [43]. The device incorporates a 650 kHz self-focusing acoustic transducer (SFAT) using PZT and PDMS air-cavity Fresnel acoustic lens (ACFAL) to deliver focused ultrasound with high spatial resolution and acoustic intensity. Robust device adhesion is achieved by incorporating 2-acrylamido-2-methylpropane sulfonic acid (AMPS) and glycerol into the bioadhesive couplant, enabling stable targeting of the primary somatosensory cortex (S1).

## 3. Current Limitations and Future Prospects

Although wearable ultrasound has enabled significant advances in health monitoring, several technical and translational challenges continue to limit its widespread clinical adoption. High power consumption remains a critical limitation in continuous deep-tissue imaging, as it requires large energy supplies and trade-offs among monitoring duration, back-end connection, and portability [43,44,45,46]. Achieving high image quality with flexible ultrasound transducers remains a significant challenge. During vigorous physical activity, substantial probe deformation causes unpredictable shifts in transducer positions and field of view, thereby introducing phase distortions, compromising beamforming precision, and degrading overall imaging performance [47,48]. While artificial intelligence (AI) mitigation has been proposed, achieving consistent clinical-grade resolution, especially for multi-dimensional or high-depth applications, remains elusive [49,50]. Annual long-term wearability also remains unexplored, as current innovation is hindered by material fatigue, piezoelectric degradation, and potential skin irritation from bioadhesive interfaces [51,52].

Future efforts in wearable ultrasound devices include low-power transducers, body-motion energy harvesting, and intelligent power management for extended operation on the skin surface [53,54,55,56,57]. Material innovations, such as fatigue-resistant piezo-composites, self-adapting encapsulants, and enhanced bioadhesives, will improve durability and long-term comfort [58]. In addition, the integration of multimodal sensing with AI-driven diagnostics can potentially improve resolution, reduce artifacts, and enable predictive analytics based on collected data [59,60,61]. Standardized, scalable manufacturing, performance optimization, and streamlined regulatory pathways will be essential to accelerate clinical translation, personalized monitoring, and global health applications.

Given future biomedical applications, the design of wearable ultrasound devices could be further optimized and manufactured. The future applications of the newly developed wearable ultrasound devices will be focused on the following areas. (1) Continuous and home-based monitoring. Conventional ultrasound imaging remains operator-dependent and confined to clinical environments. However, wearable ultrasound devices offer the longitudinal assessment of internal organ structure and function, including the heart, lungs, blood pressure, and liver. Such continuous, non-invasive monitoring enables earlier disease detection, real-time evaluation, and remote management of human health, which is fundamentally expanding the scope of preventive and decentralized healthcare. (2) Acoustic neuromodulation. Ultrasound-mediated neurostimulation enables non-invasive, spatially precise modulation of neural circuits. Theoretically, ultrasound can induce neuronal responses without surgical implantation. High-frequency wearable ultrasound devices could, in principle, achieve focal resolutions on the order of 50–70 μm, substantially exceeding that of conventional electrical stimulation while allowing prolonged or programmable stimulation paradigms. Hence, wearable devices can open new avenues for neuromodulation. (3) Access to deep tissue structures. A defining advantage of ultrasound over optical and electrical wearable technologies is its ability to access deep tissues. Whereas most current wearable sensors are limited to surface or near-surface signals, ultrasound penetrates centimeters into biological tissue, enabling dynamic visualization of cardiovascular mechanics, organ motion, and internal physiological processes. This capacity to bridge superficial form factors with deep anatomical information positions wearable ultrasound as a uniquely powerful modality for next-generation physiological monitoring. (4) Drug delivery. Therapeutically, wearable ultrasound devices can further facilitate localized drug delivery. By applying acoustic cavitation, ultrasound can enhance transdermal and tissue-specific drug penetration. Integrated with wearable systems, it could realize programmable delivery and dosing without invasive procedures, expanding opportunities in dermatology, pain management, and systemic therapy. (5) Integration with AI and Smart Systems. Finally, the wearable ultrasound devices with artificial intelligence and intelligent control systems will be of significance. AI-driven algorithms can compensate for motion artifacts and signal variability, enable automated image reconstruction and interpretation, and deliver adaptive, personalized therapeutic feedback. Collectively, these advances suggest that wearable ultrasound technologies will become multifunctional platforms that integrate continuous imaging monitoring, neuromodulation, targeted therapy, and intelligent systems, redefining the boundaries between diagnostic imaging and real-time physiological intervention.

## 4. Conclusions

Wearable ultrasound devices represent a paradigm shift toward continuous, operator-independent biomedical monitoring. Advances across strap-based, adhesive, and stretchable wearable designs have enabled real-time functional assessment in cardiovascular, hemodynamic, musculoskeletal, and emerging therapeutic domains. These novel developments unlock new opportunities for chronic disease management, rehabilitation, and personalized remote care. Although challenges persist in power efficiency, imaging fidelity, long-term reliability, and large-scale clinical validation, continued interdisciplinary efforts are expected to establish wearable ultrasound systems as a foundational technology for proactive and precision medicine.
